# Anatomical Variations of the Jugular Bulb: A Critical and Comprehensive Review

**DOI:** 10.3390/medicina60091408

**Published:** 2024-08-28

**Authors:** Răzvan Costin Tudose, Mugurel Constantin Rusu, George Triantafyllou, Maria Piagkou, Corneliu Toader, Petrinel Mugurel Rădoi

**Affiliations:** 1 Division of Anatomy, Faculty of Dentistry, “Carol Davila” University of Medicine and Pharmacy, 020021 Bucharest, Romania; razvan-costin.tudose0721@stud.umfcd.ro; 2 Department of Anatomy, School of Medicine, Faculty of Health Sciences, National and Kapodistrian University of Athens, 75 Mikras Asias str, Goudi, 11527 Athens, Greece; georgerose406@gmail.com (G.T.); mapian@med.uoa.gr (M.P.); 3 Division of Neurosurgery, Department 6—Clinical Neurosciences, Faculty of Medicine, “Carol Davila” University of Medicine and Pharmacy, 020021 Bucharest, Romania; corneliu.toader@umfcd.ro (C.T.); petrinel.radoi@umfcd.ro (P.M.R.); 4 Clinic of Neurosurgery, “Dr. Bagdasar-Arseni” Emergency Clinical Hospital, 041915 Bucharest, Romania

**Keywords:** internal jugular vein, jugular foramen, computed tomography, dehiscence, diverticulum

## Abstract

*Background and Objectives*: The jugular bulb (JB) is the uppermost part of the internal jugular vein receiving the sigmoid sinus. The aim of the present research is to aid the comprehension of the JB, its abnormalities, and surrounding structures for improving both academic and surgical awareness. *Materials and Methods*: Various studies on this topic were critically reviewed. Cone-beam CT scans and CT and MR angiograms were used to demonstrate each type of the discussed variations. *Results*: Variations in the JB anatomy were thoroughly documented: high JB, dehiscent JB, hypoplasia and hyperplasia, and diverticula of the JB, as they have significant clinical implications, particularly in the context of otological and neuro-otological surgery, skull base pathology, and diagnostic imaging. Definitions and critical arguments were also specified to clarify existing literature. Additionally, we present a case report illustrating a high and dehiscent JB, an anatomical variation of clinical interest due to its potential for misdiagnosis as a glomus tumor. Another case describes a dehiscent JB with a hypotympanic air cell protruding into it, further highlighting the variability of this condition. *Conclusions*: It is necessary to proceed with caution when observing abnormal morphological characteristics of the JB. Preoperative assessment of each case is essential for optimal outcomes.

## 1. Introduction

**Figure 1:**
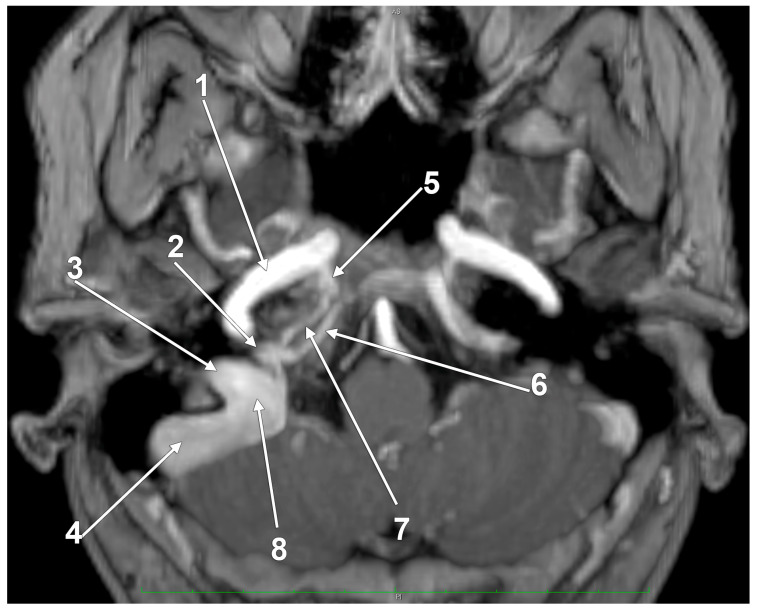
The venous anatomy antero-medial to the initial segment of the internal jugular vein. MR angiogram. Curved-planar axial slice viewed inferiorly. Right side. 1. internal carotid artery; 2. anterior condylar confluence; 3. internal jugular vein; 4. sigmoid sinus; 5. venous plexus of Rektorzik; 6. inferior petrosal sinus; 7. inferior petrooccipital vein of Trolard; 8. jugular bulb.

**Figure 2:**
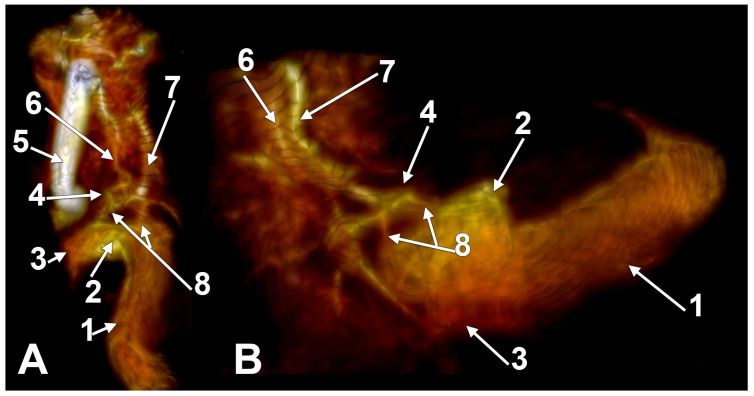
The anterior condylar confluent of Trolard. MR angiogram. Three-dimensional volume renderings on the right side, viewed supero-laterally (**A**) and laterally (**B**). The internal carotid artery was cropped out in (**B**). 1. sigmoid sinus; 2. jugular bulb; 3. internal jugular vein; 4. anterior condylar confluent; 5. internal carotid artery; 6. inferior petrooccipital vein of Trolard; 7. inferior petrosal sinus; 8. bridging veins.

The jugular bulb (JB) is a key venous structure, defined as a dome-shaped dilation that extends into the floor of the tympanum. It connects the internal jugular vein (IJV) and the sigmoid sinus and occupies the jugular fossa on the extracranial side of the jugular foramen [1]. Accurate identification and distinction between these anatomical entities are essential, denoting different anatomical features. The anatomical terminology indicates the superior bulb of the IJV (bulbus superior venae jugularis) as the dilatation of the IJV at its origin in the jugular foramen [2]. Different venous affluents empty into the JB via the anterior condylar confluent (ACC) of Trolard or the petrosal confluence. The ACC occupies the anterior condylar fossa where the hypoglossal canal opens extracranially. It is, therefore, antero-inferior to the JB. The ACC (Figure 1 and Figure 2) is connected directly with the IJV, inferior petrosal sinus, anterior condylar vein, Trolard’s inferior petrooccipital vein, lateral condylar vein [3], with the prevertebral vein [4] or plexus [5], and, eventually, with clival diploic veins [6]. These veins are devoid of valves so they allow multidirectional venous flow [3]. Moreover, the ACC is connected to the IJV or the IJV bulb by bridging veins, allowing for retrograde venous flow into the sigmoid sinus in cases of dural arteriovenous fistulas [3].

The JB variations include high JB (HJB), hypoplasia and hyperplasia, JB dehiscence, and JB diverticulum (JBD) [7,8,9,10,11]. The most prevalent and significant anatomical variant of the JB is the HJB, which frequently makes it more challenging to diagnose, plan treatments, and perform surgical procedures [7,8,10,12,13,14,15].

This review aims to thoroughly investigate the anatomical variations of the JB as described in publications. Archived computed tomography (CT) and magnetic resonance (MR) angiograms and cone-beam computed tomography (CBCT) scans from previous studies [11,16,17,18,19] were included to depict specific anatomical details. Studies on human anatomical variations of the JB were included in the review. However, studies focusing on clinical aspects of the jugular bulb, low-quality papers, or papers that could not be obtained as full-text articles were excluded. This approach ensures that the review is comprehensive yet maintains a focus on relevant anatomical data. The current scientific review aims to go beyond summarizing different studies by critically evaluating their strengths and weaknesses compared to the existing literature.

## 2. Hypoplasia and Hyperplasia of the Jugular Bulb

The literature review highlights a gap in the objective definition and quantitative analysis of JB hypoplasia and hyperplasia. For instance, two studies by Graham [9,20] that suggest average dimensions of the JB as being 15 mm wide and 20 mm high lack empirical evidence such as reference numbers, measurements, standard deviations, or range data. This omission undermines the relevance of these claims, as they are not supported by concrete data. Similarly, Friedmann’s study [21] of the prevalence of JB abnormalities and resultant inner ear dehiscence provides some measurements for the normal JB (anteroposterior dimension: 0.68 cm; mediolateral dimension: 0.76 cm) without addressing the maximum diameter. While their findings suggest that the normal JB diameter corresponds to the dimensions we propose, ranging between 0.5 and 1.5 cm, this does not encompass hyperplastic or hypoplastic conditions, leaving a gap in our understanding of JB variations beyond these parameters.

Additionally, the use of terms such as phlebectasia instead of hyperplasia without any numerical data or measurements further illustrates the ambiguity in the current academic discourse regarding JB dilatation [22]. This lack of precision renders such descriptions irrelevant in the context of identifying and classifying JB abnormalities effectively. Moretti (1976) documented a case of sinusojugular hypoplasia, without any numerical measurements to define this condition [23].

Kennedy et al.’s [24] considerations on JB enlargement also fall into this pattern of qualitative discussion without offering specific measurements or objective criteria to define JB enlargement. The focus remains on the clinical implications, variations in position and size, and their impact on otologic surgery and patient symptoms, without quantitative thresholds for what constitutes enlargement. This qualitative approach highlights the variability in JB anatomy but fails to provide a framework for systematic assessment.

It has been reported that there is a higher occurrence of enlarged JBs in individuals with achondroplasia [25]. Enlargement of the JB has also been observed in patients suffering from Paget’s disease, especially those with significant skull involvement [26]. This enlargement is primarily attributed to bone remodeling, though increased venous flow might also play a role [27]. In cases of widespread transformation of pagetoid bone, it was noted that the arteries, arterioles, and corresponding venous blood vessels significantly increase in size and number, leading to a considerable expansion of the JB in all temporal bones undergoing active bone transformation [28,29].

Furthermore, it was studied that in sclerosteosis, a significant narrowing of the jugular venous system occurs, leading to intracranial hypertension due to bone remodeling, resulting in extreme constriction of the JB [30]. It was found that the lumina of both the lower portion of the sigmoid sinus and the JB were extremely constricted [31]. Surgical intervention, including decompression of the transverse sigmoid sinus and JB, along with posterior and, if required, anterior craniectomy for decompression, were identified as potentially lifesaving measures [32].

While these studies provide relevant information about the occurrence of enlarged JBs in conditions like achondroplasia and Paget’s disease, as well as the notable narrowing seen in sclerosteosis, it is noteworthy that these analyses, despite detailing the impacts of such diseases on the dimensions of the JB, do not offer a precise categorization or definition for what constitutes an altered dimension of the JB.

**Figure 3:**
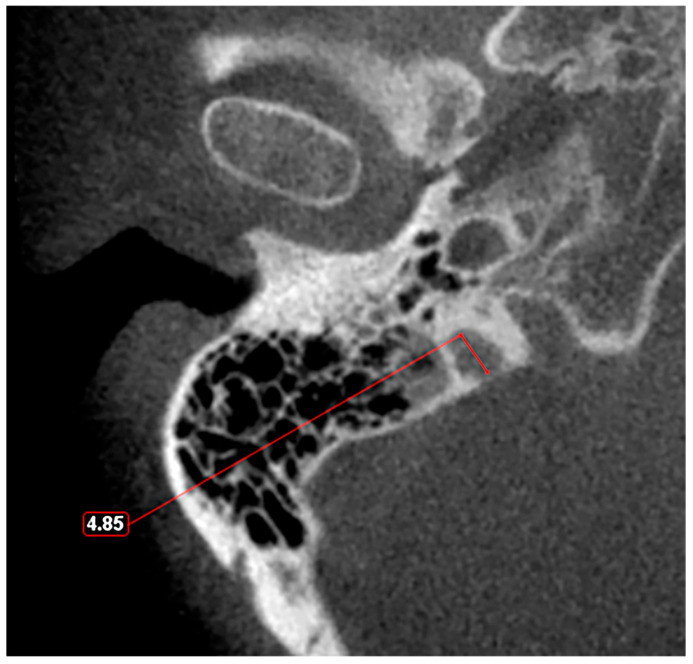
Axial slice through the right hypoplastic jugular bulb reveals an underdeveloped structure with a maximum diameter of less than 5 mm.

**Figure 4:**
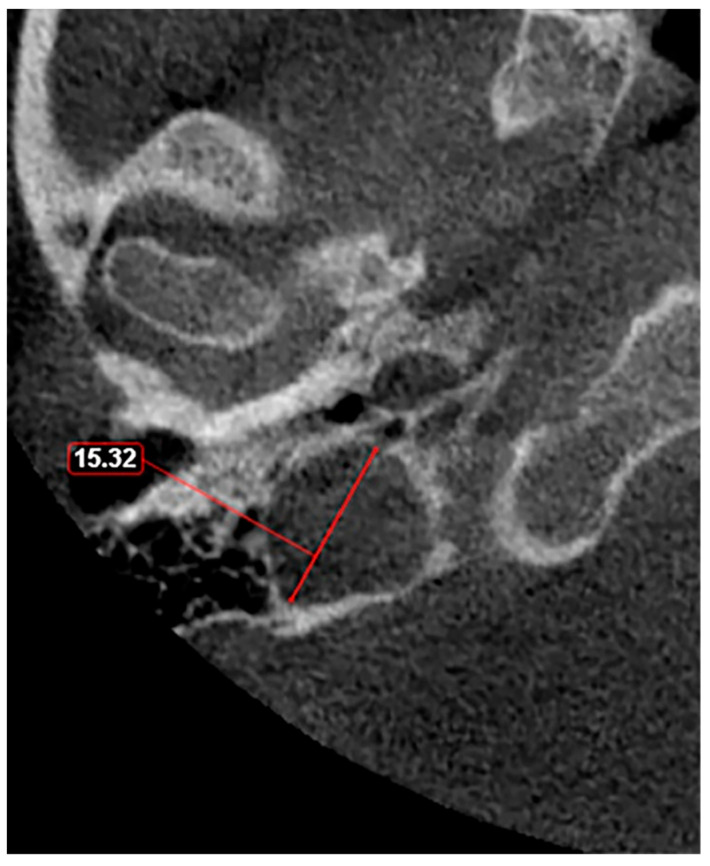
Axial slice through the right hyperplastic jugular bulb illustrates an enlarged structure with a maximum diameter exceeding 15 mm.

Given these observations, there is a clear need for a standardized classification system to accurately assess JB variants. As proposed in a previous study [11], a JB diameter of 5 mm or less should be defined as hypoplasia (Figure 3), while a JB diameter of a minimum of 15 mm should be considered as hyperplasia (Figure 4). This suggestion aims to fill the current void in literature with a simple yet effective method to categorize JB dimensions, facilitating clearer communication, diagnosis, and research in the field. The adoption of such a classification could standardize the assessment of JB variants, enhancing the accuracy of clinical and surgical planning and contributing to a more robust academic understanding of these conditions.

**Figure 5:**
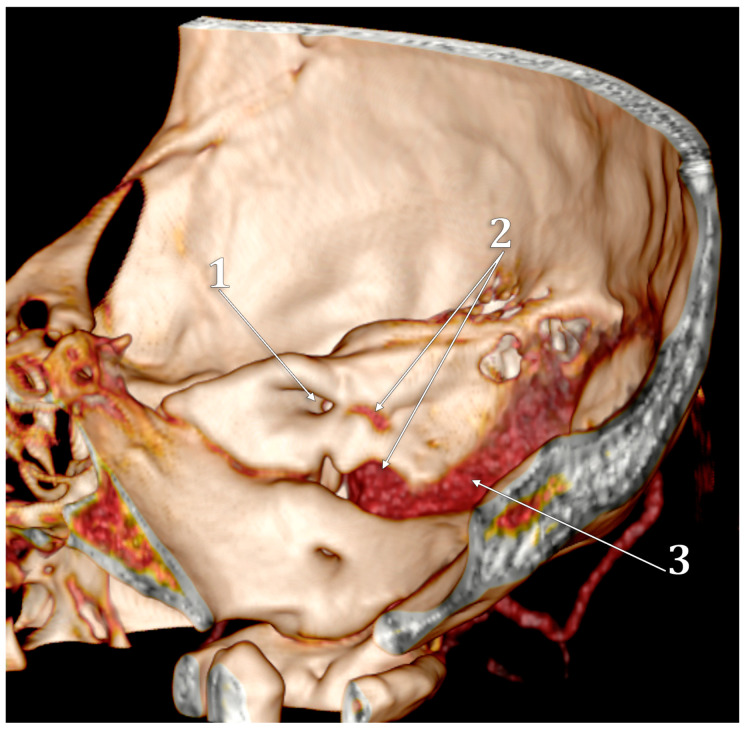
Three-dimensional volume rendering of the right temporal pyramid. Medial view. 1. internal acoustic canal; 2. high jugular bulb; 3. sigmoid sinus.

In the documented case, the right IJV had a caliber of 11.4 mm, while the left IJV measured 7.28 mm in caliber. The right JB (Figure 5) had a transverse diameter of 13.8 mm, whereas the left JB measured 8.16 mm. Although both JB values fall within the normal range of 5 to 15 mm, the dominance of the right JB and right IJV is noteworthy.

## 3. Diverticula

**Figure 6:**
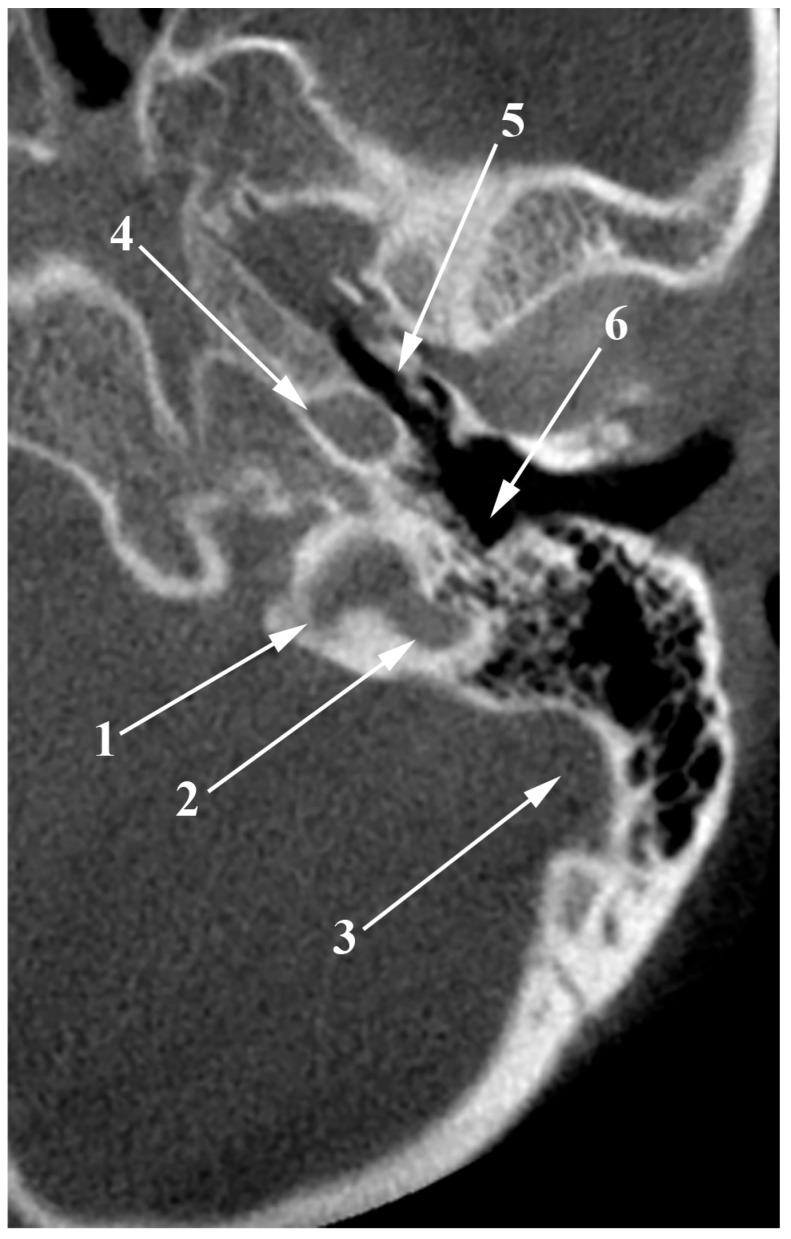
Axial slice through the left jugular bulb (JB). 1. JB; 2. posterolateral diverticulum of the JB; 3. sigmoid sinus; 4. internal carotid artery; 5. auditory tube; 6. hypotympanum.

Several studies have explored the prevalence and clinical implications of JBD, providing insights into its occurrence, anatomical definition, and potential health risks. Wadin et al. [33,34,35] conducted comprehensive research using dry skulls with plastic casts and CT scans, discovering JBD in 32 of 84 cases examined via CT and 17 out of 245 in dry skulls. Their findings highlighted that JBD typically extends in a medio-vertical direction, observed in 28 cases. According to their definition, a high fossa, which reaches up to and above the level of the round window, and a JBD is identified as a protrusion from the dome of the high fossa, delineated by a waist-like margin in the fossa’s contour. Bilgen et al. [13] reported a lower prevalence, identifying 17 cases of JBD out of 1474 CT scans. They defined JBD as a true venous anomaly, an outpouching of the JB that extends superiorly, medially, and posteriorly within the petrous bone, emphasizing the distinction between posterior and anterior JBD relative to an axial reference line. Jahrsdoerfer et al. [36] found an even lower prevalence, with only two cases among 239 radiographed patients, and Friedmann et al. [21] reported a prevalence of 3%. Atilla et al. [37] found a prevalence of 7.9%, studying 350 HRCT scans. The figures provided are of insufficient quality to clearly comprehend the methodology, rendering the results questionable in terms of reliability. In comparison to these previous results, the JBD on CBCT scans was seen in 8% of the cases [11] (Figure 6).

Despite the varying prevalence rates reported, the lack of standardized criteria for defining outpouchings as JBD based on their size relative to the main vessel highlights a gap in the literature, necessitating further studies. The size and shape of these JBD can vary significantly, from tall and slender to broad and clumsy, further complicating consistent classification [33].

Clinical implications of JBD are significant, ranging from inadvertent encounters during surgery leading to hemorrhage, as reported by Graham (1975) [9], to conductive hearing loss and pulsatile tinnitus for lateral JBD, and vertigo, pulsatile tinnitus, and sensorineural hearing loss for medial types, as described by Presutti et al. [38]. Diagnostic and management dilemmas are discussed by Pappas et al. [39], who emphasize the importance of HRCT in the diagnosis and management of petrous jugular malposition (diverticulum). El-Kashlan et al. [40] note that while a large JB without significant bone remodeling is common, the JBD is a rare venous anomaly, with their review adding to the discourse by highlighting JBD’s potential to cause pulsatile tinnitus.

### The “Condylar Jugular Diverticulum”: True or False?

**Figure 7:**
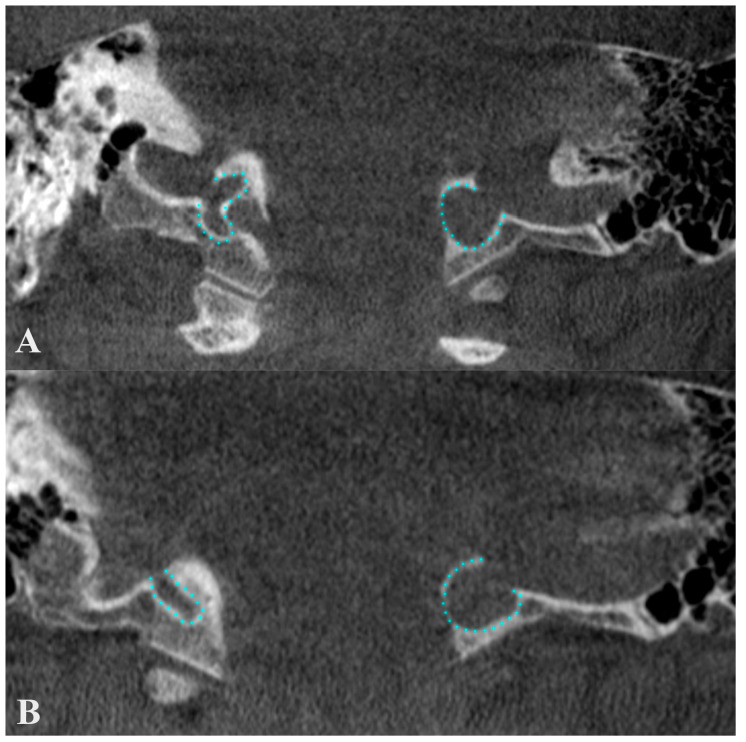
Anterior coronal plane (**A**) through the conjoining of the sigmoid sinus with the internal jugular vein and posterior (**B**), through condylar canals, outlined with blue. The figure highlights the erroneous information about a condylar diverticulum of the jugular bulb, as the outpouchings represent condylar canals that extend posteriorly from the jugular foramen to the condylar fossa.

The terminology “condylar jugular diverticulum” [41,42,43] inaccurately describes the anatomical feature observed. Instead of identifying a diverticulum, the correct interpretation involves a notably enlarged condylar canal that communicates directly with the JB from the condylar fossa. This canal serves as a conduit for emissary veins, establishing a venous connection between the JB and the suboccipital venous plexus (Figure 7A). Upon examination in a posterior coronal plane (Figure 7B), these canals are still visible posterior to the junction of the JB and sigmoid sinus, maintaining their caliber and extending to their openings in the condylar fossa. Since none of the above-mentioned studies provided posterior coronal sections to assess the condylar canals, it is imprecise to affirm the reported structure is a JBD. This clarification is crucial because it corrects the misclassification and shows that the structures displayed are, in fact, either significantly enlarged (left side) or standard (right side) condylar canals, not JBD.

## 4. High Jugular Bulb

Previous studies that assessed this variation are summarized in Table 1.

First, it is important to differentiate between an HJB and a JBD. Venography provided a clear distinction between these two, emphasizing the significance of imaging techniques in accurately identifying these conditions [20]. Wadin et al. defined an HJB as a waist-like configuration of the JB [33]. However, as suggested by Vachata et al. [14], the JBD is better characterized as a finger-like protrusion. Therefore, we highlight the distinction between superior diverticula and a genuine HJB, especially in terms of their relative size to the primary vessel. Friedmann et al. [21] further elaborates that HJBs can be differentiated from JBD using temporal bone CT, as opposed to temporal bone histological specimens. This methodology underscores the critical role of advanced imaging in the precise anatomical classification of JB variations, facilitating accurate diagnoses and informing surgical planning. Manjila et al.’s [58] introduction of a novel classification system for JBs, although not providing prevalence data, highlights the ongoing effort to better categorize these anatomical variations. They proposed a CT–based 5-type classification of the anatomical location of the JB (Table 2). However, the absence of specific prevalence data or detailed measurements in their classification system points to the necessity for more numerical evidence to validate any new categorization framework.

**Figure 8:**
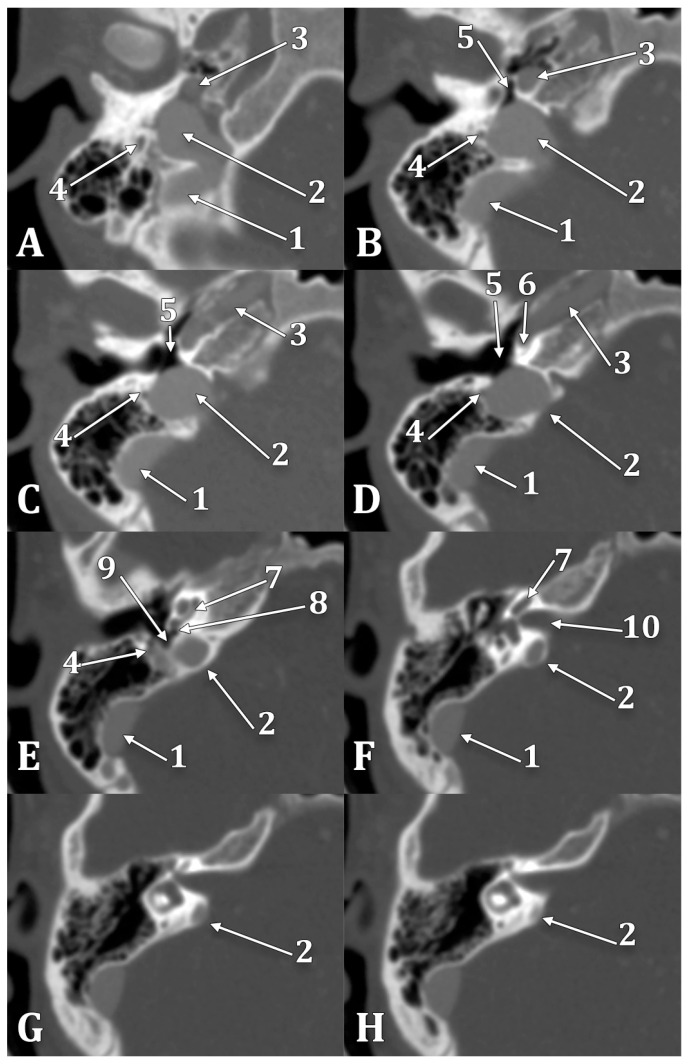
Inferior-to-superior sequence (**A**–**H**) of axial slices through the right jugular bulb (JB), inferiorly viewed. 1. sigmoid sinus; 2. JB; 3. internal carotid artery; 4. facial nerve; 5. hypotympanum; 6. fustis; 7. basal turn of cochlea; 8. round window; 9. sinus tympani; 10. internal acoustic canal.

In the reported case, a high and dehiscent JB was documented, as seen in Figure 5. Posterior and postero-laterally, a 0.6 mm thin wall separated the right JB from mastoid air cells; the internal wall of the canal of the facial nerve was also dehiscent towards the JB. Anterolaterally, the right JB was dehiscent towards the retro- and hypotympanum and neighbored the fustis, round foramen, and sinus tympani (Figure 8). The superior side of the JB reached inferiorly to the cochlea and projected a superior diverticulum length of 3.11 mm posterior to the IAC, reaching 1.33 mm posterior to the IAC’s roof. It had a thin posteromedial wall and protruded onto the posterior face of the temporal pyramid in the posterior cranial fossa. On the lateral side of the JB and IJV, a 1.17 cm long and 1.58 mm thin right styloid process was also documented. This anatomical variant is clinically significant due to its potential misdiagnosis as a glomus tumor [59], making preoperative imaging essential.

The left JB reached superiorly at 1.17 mm beneath the PSC. It also reached internally to the sinus tympani posterior and the round window. The upper end of the left JB reached 7.82 mm posterior to the floor of the IAC. There were no dehiscent walls of the left JB.

**Figure 9:**
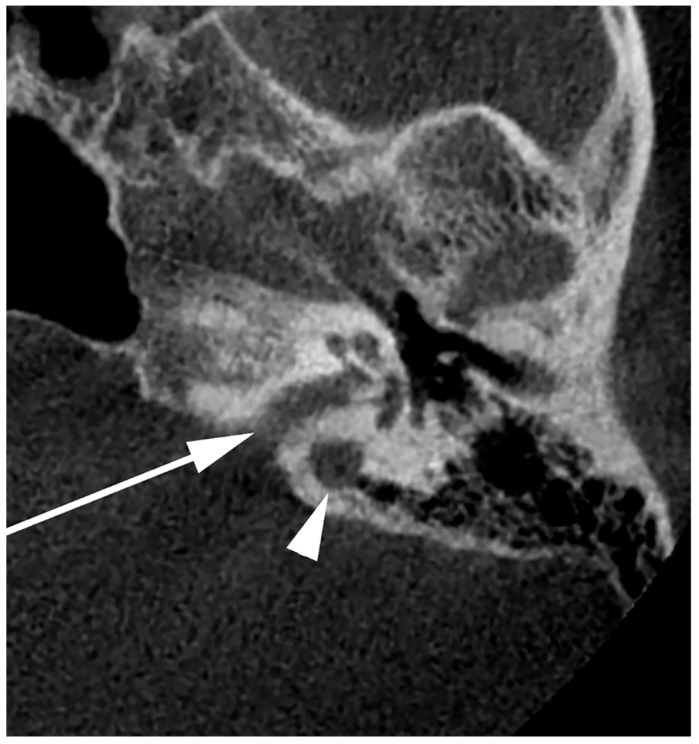
Axial slice through the right internal acoustic canal (IAC) (arrow). A high jugular bulb can be identified postero-laterally to the IAC (arrowhead).

During posterior transpetrosal approaches targeting the posterior fossa, surgeons open the dura mater on the temporal bone’s posterior aspect, which interfaces with the cerebellopontine angle. This operative zone, known as Trautmann’s triangle (TT), delineated by the JB at its inferior vertex, the sino-dural angle forming its superior base, and the posterior semicircular canal marking its anterior boundary, is pivotal in surgical interventions like posterior petrosectomy [60,61]. TT’s anatomical significance is underscored by the proximity of the JB to the posterior semicircular canal, with distances ranging from 6 to 11 mm, averaging 8.5 mm [62]. An HJB reaching or surpassing the level of the IAC (Figure 9) presents a critical surgical variability [63]. It potentially restricts the surgical field within TT due to dimensional reduction, thereby elevating the risk of JB injury during surgery [46,53]. This anatomical variation significantly influences surgical planning and execution, necessitating detailed preoperative assessment to minimize complications and optimize patient outcomes.

Among the symptoms of an HJB, the most common is tinnitus [64], followed by vertigo, pre-syncope, and hearing disturbances [15,65]. Managing an HJB during middle ear surgery is challenging. A postauricular approach has been previously recommended due to its broader surgical field, allowing the surgeon to navigate from the facial recess through the round window niche to the hypotympanum and, therefore, reducing the risk of injuring the JB [66]. If hemorrhage occurs, bleeding control can be achieved by immediately packing with various hemostatic agents [20,67,68]. Thus, awareness of this anatomical variation through preoperative imaging is essential, as the HJB is not a contraindication for middle ear surgery [66].

## 5. Dehiscent Jugular Bulb

**Figure 10:**
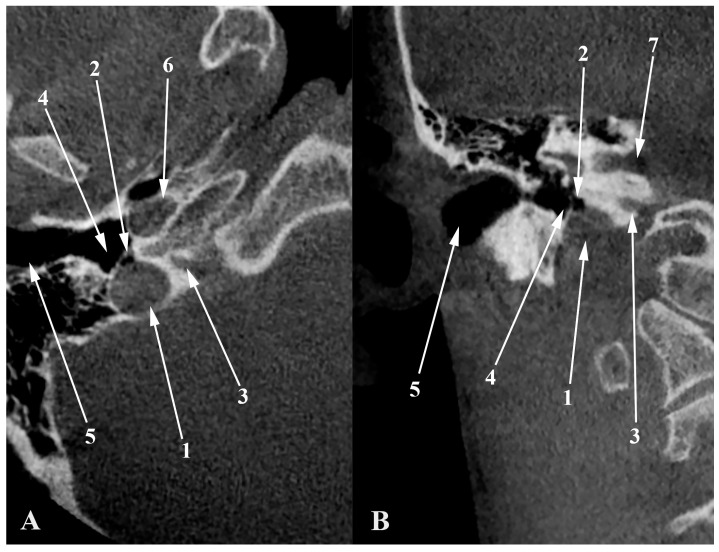
Axial (**A**) and coronal (**B**) views through the jugular bulb. 1. dehiscent JB; 2. air cell from the hypotympanum (hypotympanic cell) protruding into the JB; 3. cochlear aqueduct; 4. hypotympanum; 5. external acoustic canal (cartilaginous part); 6. carotid canal; 7. internal acoustic canal.

A dehiscent JB (Figure 8) occurs when the JB extends into the middle ear cavity through a dehiscent sigmoid plate, lacking a complete bony covering and being covered only by a thin mucosal layer [67,69]. This anatomical variant is particularly susceptible to surgical injury [67]. Atilla et al. found that HJBs were also dehiscent in 27 cases out of 142; 74% occurred on the right side, 26% on the left, and 7% were bilateral [37]. Wang et al. reported a significant correlation between carotid canal dehiscence and HJBs, noting that the latter is often associated with a thinner carotid canal wall [57]. It is, therefore, reasonable to expect that an HJB could have an overall deficiency in surrounding bone. In Crouzon’s disease patients, approximately 55% of the JBs (12 out of 22) were either protruding or dehiscent, posing a high risk of puncture during myringotomy, which underscores the necessity for preoperative imaging [70]. Tomura et al. presented a prevalence of 2.4% of the dehiscent HJB [71], while Sayit et al. reported a prevalence of 3.5% [15]. Another study indicated a 7.5% prevalence of dehiscent JBs, identified by the lack of a hyperdense bony septum between the tympanic cavity and the JB [12]. Dehiscent JBs were also described in 4 temporal bones out of 402 analyzed, indicating a prevalence of roughly 1% [48]. Figure 10 illustrates a dehiscent JB, where an air cell from the hypotympanum (hypotympanic cell, according to a previous classification [11]) extends into the JB, opposite to the common definition. Anatomical definitions should be viewed with flexibility, as individual variations are common. Overall, the incidence of dehiscent JBs varies between 1% and 7%, reflecting the diversity and clinical challenges of this anatomical feature [10].

## 6. Jugular Bulb Variations across Age and Demographic Groups

In one study, Low et al. [72] showed that there were clear ethnic differences in the anatomical location of the HJB. They suggested that in the Caucasian subjects, the HJB usually had a more lateral location, while in Chinese subjects, it was more medially located. Of importance, this study also noted that the influence of ethnicity did not seem to affect either JB height or its location in the sagittal plane but did have an important effect on whether an HJB was likely to be medial or lateral to the cochlea. This is clinically significant, as the positional variation of the JB may be relevant in surgical planning and risk assessment, specifically with procedures concerning the temporal bone and structures related to it [20,46,72]. Moreover, Yagi et al. [73] analyzed Japanese temporal bones and reported a prevalence of 62% of the JBs as being located medially to the cochlea. This result is consistent with that of Low et al., as Japanese and Chinese populations are genetically related [74,75].

Previous studies have shown that JB size and height are age-related [21,46,57,76,77]. Wang et al. [57] conducted a study that showed age-related patterns in the occurrence and characteristics of the HJB. They found the prevalence rate for HJBs was the highest in the age group of individuals between 35 and 45 years (17.6%). Although a decreasing tendency in its existence with increasing age was noted, it remained relatively common among those above 65 years old at 12.8% (*p* = 0.039). The study also showed a significant lateral difference in JB size with increasing age: the size of the left JB decreased significantly with age (from 47.4 mm^2^ to 42.5 mm^2^), but the right JB, which had a higher prevalence of HJBs and was larger in general compared to the left, did not present the same decrease in size correlated with the patient’s age. These findings highlight that, during the clinical evaluation of the JB, not only the age but also the lateral differences should be kept in consideration. Rauch et al. [46] also noted age-related variability of the JB. Specifically, for subjects less than 6 years, HJBs were found in 6% of these cases (2 of 32), which is significantly less compared to the much higher prevalence rate of 63% (47 of 75) in older age groups (*p* < 0.01). This significant difference indicates that prevalence rates for high JBs rapidly and progressively increase with age after early childhood. Based on Friedmann et al. [21], JB abnormalities occur in 10% to 15% of patients. These abnormalities are rare in the first decade of life (<2%) but gradually increase in frequency over the first four decades, stabilizing at around 10% after age 50. Interestingly, cases of HJB have been identified in patients as young as 5 years, suggesting that some JB abnormalities are either congenital or acquired early in life, while the majority develop over the first five decades. The incidence of these abnormalities notably doubles during adolescence (ages 11–20), likely linked to pubertal growth, and experiences another significant increase between ages 31 and 50, though the cause of this is unclear. After age 50, the incidence stabilizes, indicating that few JBAs are acquired later in life. This pattern aligns with another developmental study [76] showing that the JB first appears at age 2, grows over the first 40 years, and then stabilizes in size, indicating that JB abnormalities are primarily acquired during the growth phase of the JB.

## 7. Current Limitations and Future Perspectives

As previously stated [11], the field of JB variational anatomy still requires greater standardization and deeper knowledge. First, it is important that authors should try to elaborate unique classification systems and definitions for all the above-mentioned variations to avoid further misunderstandings and to facilitate both academic comprehension of this anatomical region and research advancement in this field. This represents the first step towards standardized protocols for the management of these conditions, consistent diagnostics, and improved outcomes of their treatment. Second, the widespread adoption of imaging techniques that allow for proper evaluation of the JB and related vascular structures, such as MRI, MR, and CT angiography, is equally important for a thorough understanding of the anatomical region.

## 8. Conclusions

It is important to proceed cautiously when identifying anomalous JB characteristics. A comprehensive preoperative evaluation is necessary to correctly detect these anomalies in each individual. The structure and variations of the JB should be seen in detail using advanced imaging methods, which enable customized surgical plans, further reducing risks and guaranteeing the best possible results. Clinicians in otolaryngology and related fields can increase patient safety and treatment efficacy by identifying and comprehending the individual heterogeneity of JB anatomy.

## Figures and Tables

**Table 1:** Studies reporting prevalences of the high jugular bulbs, alongside their methodology and definition of this anatomical variation. JB—jugular bulb, IAC—internal acoustic canal, HJB—high JB, HRCT—high-resolution CT, CBCT—cone-beam CT.

Author	Definition of HJB	Method	Prevalence of HJB (%)
Singla et al. [44]	Vertical IAC–JB distance less than 2 mm	Cadavers	24.1
Vachata et al. [14]	The apex of JB reaches/traverses the floor of IAC	Cadavers/HRCT	15
Aslan et al. [45]	JB occupies more than two-thirds of the distance between IAC and JB	Dry skulls	23
Atilla et al. [37]	Extends over the level of the round window and basal turn of the cochlea	HRCT	20.3
Rauch et al. [46]	Maximum 2 mm between IAC and JB	Dry skulls	46
Hourani et al. [47]	Extends above the superior tympanic annulus	CT	33.5
Koesling et al. [48]	High location (over the plane of the basal turn of the cochlea)	HRCT	6
Roche et al. [49]	JB dome less than 6.5 mm below IAC	CT	25
Overton et al. [50]	Extended into the middle ear above the inferior rim of the bony annulus	Histologically prepared temporal bones	6
Inal et al. [51]	JB over the inferior surface of the bony annulus or basal turn of the cochlea	HRCT	6–20
Friedmann et al. [21]	JB rising above the level of the IAC in axial section	CT scans/Temporal bone histopathology	8.5/8.2
Saleh et al. [52]	JB in actual direct contact with or even lying behind the internal auditory canal	Intraoperative	4
Shao et al. [53]	Grade I, jugular bulb situated less than 1.5 mm above the low border of IAC Grade II, jugular bulb between 1.5 and 3.0 mm above the low border of the IAC Grade III, jugular bulb > 3 mm above the low border of IAC	HRCT	9
Juelke et al. [54]	Upper than IAC floor as type 4 in Manjila classification	HRCT/3D models	0
Aksoy et al. [55]	The study defines HJB as the jugular bulb lying higher than the inferior portion of the internal acoustic canal	CT	42.8
Sayit et al. [15]	The apex of the jugular foramen above the inferior bony annulus of the tympanic membrane	CT	22
Woo et al. [56]	The apex of the jugular foramen was above the inferior bony annulus of the tympanic membrane	CT	9.5
Wadin et al. [35]	A HJB reaches up to and above the level of the round window	Temporal bone plastic casts	24
Atmaca et al. [12]	JB position above the lower level of IAC	HRCT	15.2
Wang et al. [57]	A JB that lies higher than the inferior aspect of the IAC	MRI	14.5
Tudose et al. [11]	The apex of JB reaches/traverses the floor of IAC	CBCT	2

**Table 2:** Types and subtypes of the jugular bulb (JB)—the classification of Manjila et al. [58]. PSC—posterior semicircular canal; IAC—internal acoustic canal.

Type	Subtype	Description
1		no JB
2		JB below the PSC
	2A	the JB is not dehiscent into the middle ear
	2B	the JB is dehiscent into the middle ear
3		JB between the PSC and IAC
	3A	the JB is not dehiscent into the middle ear
	3B	the JB is dehiscent into the middle ear
4		JB above the IAC
	4A	the JB is not dehiscent into the IAC
	4B	the JB is dehiscent into the IAC
5		combination of dehiscences

## Data Availability

Data are available from authors on reasonable request.

## References

[1] Gray H., Standring S., Anand N., Birch R., Collins P., Crossman A., Gleeson M., Jawaheer G., Smith A.L., Spratt J.D. (2016). Gray’s Anatomy: The Anatomical Basis of Clinical Practice.

[2] Feneis H., Dauber W. (2000). Pocket Atlas of Human Anatomy: Based on the International Nomenclature.

[3] Spittau B., Millan D.S., El-Sherifi S., Hader C., Singh T.P., Motschall E., Vach W., Urbach H., Meckel S. (2015). Dural arteriovenous fistulas of the hypoglossal canal: Systematic review on imaging anatomy, clinical findings, and endovascular management. J. Neurosurg..

[4] Yamada H., Mizutani K., Akiyama T., Toda M. (2022). Extracranial prevertebral venous network of the craniocervical junction: CT-digital subtraction venography analysis. Neuroradiology.

[5] Takemoto K., Tateshima S., Rastogi S., Gonzalez N., Jahan R., Duckwiler G., Vinuela F. (2013). Onyx embolization of anterior condylar confluence dural arteriovenous fistula. BMJ Case Rep..

[6] Mortazavi M.M., Tubbs R.S., Riech S., Verma K., Shoja M.M., Zurada A., Benninger B., Loukas M., Cohen Gadol A.A. (2012). Anatomy and pathology of the cranial emissary veins: A review with surgical implications. Neurosurgery.

[7] Redfern R.E., Brown M., Benson A.G. (2014). High jugular bulb in a cohort of patients with definite Meniere’s disease. J. Laryngol. Otol..

[8] Fujimoto C., Ito K., Ishimoto S., Iwasaki S. (2007). Large jugular bulb diverticulum invading the internal auditory canal. Ann. Otol. Rhinol. Laryngol..

[9] Graham M.D. (1975). The jugular bulb. Its anatomic and clinical considerations in contemporary otology. Arch. Otolaryngol..

[10] Ball M., Elloy M., Vaidhyanath R., Pau H. (2010). Beware the silent presentation of a high and dehiscent jugular bulb in the external ear canal. J. Laryngol. Otol..

[11] Tudose R.C., Rusu M.C., Triantafyllou G., Piagkou M., Moraru L., Dumitru C.C. (2024). Jugular bulb anatomical variations and pneumatization patterns: A comprehensive CBCT analysis. Surg. Radiol. Anat..

[12] Atmaca S., Elmali M., Kucuk H. (2014). High and dehiscent jugular bulb: Clear and present danger during middle ear surgery. Surg. Radiol. Anat..

[13] Bilgen C., Kirazli T., Ogut F., Totan S. (2003). Jugular bulb diverticula: Clinical and radiologic aspects. Otolaryngol. Head Neck Surg..

[14] Vachata P., Petrovicky P., Sames M. (2010). An anatomical and radiological study of the high jugular bulb on high-resolution CT scans and alcohol-fixed skulls of adults. J. Clin. Neurosci..

[15] Sayit A.T., Gunbey H.P., Fethallah B., Gunbey E., Karabulut E. (2016). Radiological and audiometric evaluation of high jugular bulb and dehiscent high jugular bulb. J. Laryngol. Otol..

[16] Rusu M.C., Tudose R.C., Vrapciu A.D., Popescu S.A. (2024). Lowered hyoid bone overlapping the thyroid cartilage in CT angiograms. Surg. Radiol. Anat..

[17] Triantafyllou G., Uchino A., Vassiou K., Tudose R.C., Rusu M.C., Vlychou M., Tsakotos G., Piagkou M. (2024). Fenestration of the anterior and posterior cerebral arteries in coexistence with a contralateral posterior cerebral artery of fetal origin. Surg. Radiol. Anat..

[18] Rusu M.C., Lazar M., Vrapciu A.D. (2023). Bihemispheric Right Anterior Cerebral Artery, Fenestrated Origin of the Left Pericallosal Artery, Fenestrated Basilar Artery, Double Right Posterior Cerebral Artery. J. Craniofac. Surg..

[19] Rusu M.C., Lazar M., Toader C. (2023). False Absence of the Anterior Communicating Artery and a Median Artery of Corpus Callosum. J. Craniofac. Surg..

[20] Graham M.D. (1977). The jugular bulb: Its anatomic and clinical considerations in contemporary otology. Laryngoscope.

[21] Friedmann D.R., Eubig J., Winata L.S., Pramanik B.K., Merchant S.N., Lalwani A.K. (2012). Prevalence of jugular bulb abnormalities and resultant inner ear dehiscence: A histopathologic and radiologic study. Otolaryngol. Head Neck Surg..

[22] Walsh R.M., Lannigan F.J., McGlashan J.A., Bowdler D.A. (1993). Jugular bulb phlebectasia. Int. J. Pediatr. Otorhinolaryngol..

[23] Moretti J.A. (1976). Highly placed jugular bulb and conductive deafness secondary to sinusojugular hypoplasia. Arch. Otolaryngol..

[24] Kennedy D.W., el-Sirsy H.H., Nager G.T. (1986). The jugular bulb in otologic surgery: Anatomic, clinical, and surgical considerations. Otolaryngol. Head Neck Surg..

[25] West J.M., Bandy B.C., Jafek B.W. (1974). Aberrant jugular bulb in the middle ear cavity. Arch. Otolaryngol..

[26] Tamari M. (1942). XVII Histopathologic Changes of the Temporal Bone in Paget’s Disease. Ann. Otol. Rhinol. Laryngol..

[27] Nager G.T. (1975). Paget’s Disease of the Temporal Bone. Ann. Otol. Rhinol. Laryngol..

[28] Amroliwalla F.K., Bennett R.M. (1971). An Unusual Complication of Paget’s Disease. J. R. Army Med. Corps.

[29] Culebras A., Feldman R.G., Fager C.A. (1974). Hydrocephalus and dementia in Paget’s disease of the skull. J. Neurol. Sci..

[30] Nager G.T., Hamersma H. (1986). Sclerosteosis involving the temporal bone: Histopathologic aspects. Am. J. Otolaryngol..

[31] Nager G.T., Stein S.A., Dorst J.P., Holliday M.J., Kennedy D.W., Diehn K.W., Jabs E.W. (1983). Sclerosteosis involving the temporal bone: Clinical and radiologic aspects. Am. J. Otolaryngol..

[32] Hill S.C., Stein S.A., Dwyer A., Altman J., Dorwart R., Doppman J. (1986). Cranial CT findings in sclerosteosis. AJNR Am. J. Neuroradiol..

[33] Wadin K., Wilbrand H. (1986). The jugular bulb diverticulum. A radioanatomic investigation. Acta Radiol. Diagn..

[34] Wadin K., Wilbrand H. (1986). The topographic relations of the high jugular fossa to the inner ear. A radioanatomic investigation. Acta Radiol. Diagn..

[35] Wadin K., Thomander L., Wilbrand H. (1986). Effects of a high jugular fossa and jugular bulb diverticulum on the inner ear. A clinical and radiologic investigation. Acta Radiol. Diagn..

[36] Jahrsdoerfer R.A., Cail W.S., Cantrell R.W. (1981). Endolymphatic duct obstruction from a jugular bulb diverticulum. Ann. Otol. Rhinol. Laryngol..

[37] Atilla S., Akpek S., Uslu S., Ilgit E.T., Isik S. (1995). Computed tomographic evaluation of surgically significant vascular variations related with the temporal bone. Eur. J. Radiol..

[38] Presutti L., Laudadio P. (1991). Jugular bulb diverticula. ORL J. Otorhinolaryngol. Relat. Spec..

[39] Pappas D.G., Hoffman R.A., Cohen N.L., Holliday R.A., Pappas D.G. (1993). Petrous jugular malposition (diverticulum). Otolaryngol. Head Neck Surg..

[40] El-Kashlan H.K., Arts H.A., Gebarski S. (2000). Jugular diverticulum: Clinical significance. Otolaryngol. Head Neck Surg..

[41] Raghuram K., Cure J.K., Harnsberger H.R. (2009). Condylar jugular diverticulum. J. Comput. Assist. Tomogr..

[42] Jagtap R., Wazzan T., Hansen M., Kashtwari D. (2019). Condylar jugular diverticulum: A report of 3 cases. Imaging Sci. Dent..

[43] Parillo M., Vaccarino F., Mallio C.A., Quattrocchi C.C. (2023). Right Condylar Jugular Diverticulum: Contrast-enhanced Computed Tomography Findings of a Rare Anatomical Variant of Jugular Bulb. Indian J. Otolaryngol. Head Neck Surg..

[44] Singla A., Gupta T., Sahni D., Aggarwal A., Gupta A. (2016). High jugular bulb: Different osseous landmarks and their clinical implications. Surg. Radiol. Anat..

[45] Aslan A., Falcioni M., Russo A., De Donato G., Balyan F.R., Taibah A., Sanna M. (1997). Anatomical considerations of high jugular bulb in lateral skull base surgery. J. Laryngol. Otol..

[46] Rauch S.D., Xu W.Z., Nadol J.B. (1993). High jugular bulb: Implications for posterior fossa neurotologic and cranial base surgery. Ann. Otol. Rhinol. Laryngol..

[47] Hourani R., Carey J., Yousem D.M. (2005). Dehiscence of the jugular bulb and vestibular aqueduct: Findings on 200 consecutive temporal bone computed tomography scans. J. Comput. Assist. Tomogr..

[48] Koesling S., Kunkel P., Schul T. (2005). Vascular anomalies, sutures and small canals of the temporal bone on axial CT. Eur. J. Radiol..

[49] Roche P.H., Moriyama T., Thomassin J.M., Pellet W. (2006). High jugular bulb in the translabyrinthine approach to the cerebellopontine angle: Anatomical considerations and surgical management. Acta Neurochir..

[50] Overton S.B., Ritter F.N. (1973). A high placed jugular bulb in the middle ear: A clinical and temporal bone study. Laryngoscope.

[51] Inal M., Muluk N.B., Dag E., Arikan O.K., Kara S.A. (2015). The Pitfalls and Important Distances in Temporal Bone HRCT of the Subjects with High Jugular Bulbs—Preliminary Report. Adv. Clin. Exp. Med..

[52] Saleh E.A., Aristegui M., Taibah A.K., Mazzoni A., Sanna M. (1994). Management of the high jugular bulb in the translabyrinthine approach. Otolaryngol. Head Neck Surg..

[53] Shao K.N., Tatagiba M., Samii M. (1993). Surgical management of high jugular bulb in acoustic neurinoma via retrosigmoid approach. Neurosurgery.

[54] Juelke E., Butzer T., Yacoub A., Wimmer W., Caversaccio M., Anschuetz L. (2023). Assessment of jugular bulb variability based on 3D surface models: Quantitative measurements and surgical implications. Surg. Radiol. Anat..

[55] Aksoy S.H., Yurdaisik I. (2023). High riding jugular bulb: Prevalence and significance in asymptomatic children. Acta Radiol..

[56] Woo C.K., Wie C.E., Park S.H., Kong S.K., Lee I.W., Goh E.K. (2012). Radiologic analysis of high jugular bulb by computed tomography. Otol. Neurotol..

[57] Wang J., Feng Y., Wang H., Li C., Wu Y., Shi H., Yin S., Chen Z. (2020). Prevalence of High Jugular Bulb across Different Stages of Adulthood in A Chinese Population. Aging Dis..

[58] Manjila S., Bazil T., Kay M., Udayasankar U.K., Semaan M. (2018). Jugular bulb and skull base pathologies: Proposal for a novel classification system for jugular bulb positions and microsurgical implications. Neurosurg. Focus.

[59] Phelps P.D., Lloyd G.A. (1986). Vascular masses in the middle ear. Clin. Radiol..

[60] Sincoff E.H., McMenomey S.O., Delashaw J.B. (2007). Posterior transpetrosal approach: Less is more. Neurosurgery.

[61] Miller C.G., van Loveren H.R., Keller J.T., Pensak M., el-Kalliny M., Tew J.M. (1993). Transpetrosal approach: Surgical anatomy and technique. Neurosurgery.

[62] Tubbs R.S., Griessenauer C., Loukas M., Ansari S.F., Fritsch M.H., Cohen-Gadol A.A. (2014). Trautmann’s triangle anatomy with application to posterior transpetrosal and other related skull base procedures. Clin. Anat..

[63] Alonso F., Dekker S.E., Wright J., Wright C., Alonso A., Carmody M., Tubbs R.S., Bambakidis N.C. (2017). The Retrolabyrinthine Presigmoid Approach to the Anterior Cerebellopontine Region: Expanding the Limits of Trautmann Triangle. World Neurosurg..

[64] Weiss R.L., Zahtz G., Goldofsky E., Parnes H., Shikowitz M.J. (1997). High jugular bulb and conductive hearing loss. Laryngoscope.

[65] El-Begermy M.A., Rabie A.N. (2010). A novel surgical technique for management of tinnitus due to high dehiscent jugular bulb. Otolaryngol. Head Neck Surg..

[66] Huang B.R., Wang C.H., Young Y.H. (2006). Dehiscent high jugular bulb: A pitfall in middle ear surgery. Otol. Neurotol..

[67] Moore P.J. (1994). The high jugular bulb in ear surgery: Three case reports and a review of the literature. J. Laryngol. Otol..

[68] Couloigner V., Grayeli A.B., Bouccara D., Julien N., Sterkers O. (1999). Surgical treatment of the high jugular bulb in patients with Meniere’s disease and pulsatile tinnitus. Eur. Arch. Otorhinolaryngol..

[69] Harnsberger R., Hudgens P., Wiggins R., Davidson C. (2006). Dehiscent jugular bulb. Diagnostic Imaging: Head and Neck.

[70] van Die A., de Groot J.A., Zonneveld F.W., Vaandrager J.M., Beck F.J. (1995). Dehiscence of the jugular bulb in Crouzon’s disease. Laryngoscope.

[71] Tomura N., Sashi R., Kobayashi M., Hirano H., Hashimoto M., Watarai J. (1995). Normal variations of the temporal bone on high-resolution CT: Their incidence and clinical significance. Clin. Radiol..

[72] Low W.K., Fenton J.E., Fagan P.A., Gibson W.P. (1995). The influence of race on the position of the jugular bulb. J. Laryngol. Otol..

[73] Yagi M. (1992). [A temporal bone study of the jugular fossa]. Nihon Jibiinkoka Gakkai Kaiho.

[74] Wang Y., Lu D., Chung Y.J., Xu S. (2018). Genetic structure, divergence and admixture of Han Chinese, Japanese and Korean populations. Hereditas.

[75] Horai S., Murayama K., Hayasaka K., Matsubayashi S., Hattori Y., Fucharoen G., Harihara S., Park K.S., Omoto K., Pan I.H. (1996). mtDNA polymorphism in East Asian Populations, with special reference to the peopling of Japan. Am. J. Hum. Genet..

[76] Friedmann D.R., Eubig J., McGill M., Babb J.S., Pramanik B.K., Lalwani A.K. (2011). Development of the jugular bulb: A radiologic study. Otol. Neurotol..

[77] Okudera T., Huang Y.P., Ohta T., Yokota A., Nakamura Y., Maehara F., Utsunomiya H., Uemura K., Fukasawa H. (1994). Development of posterior fossa dural sinuses, emissary veins, and jugular bulb: Morphological and radiologic study. AJNR Am. J. Neuroradiol..

